# Supplementary material for: Ceruloplasmin-Deficient Mice Show Dysregulation of Lipid Metabolism in Liver and Adipose Tissue Reduced by a Protein Replacement
Source: Int J Mol Sci. 2023 Jan 6;24(2):1150. doi: 10.3390/ijms24021150 (PMC9863737; doi:10.3390/ijms24021150)
Supplement: Supplementary file 1 [file ijms-24-01150-s001.zip › ijms-1976661-supplementary.pdf]

Supplementary Materials

Ceruloplasmin-deficient mice show lipid metabolism dysregulation in liver and adipose tissue reduced by the protein replacement

Raia S, Conti A, Zanardi A, Ferrini B, Scotti MG, Gilberti E, De Palma G, David S, Alessio M

Inventory of Supplementary Materials:

Supplementary Material Figures S1 – S5

Supplementary Materials Table S1

Supplementary Material Figure S1

<u>Analysis of ceruloplasmin expression level in liver and perigonadal adipose tissue in C57Bl/6J wild-type mice</u>

Western blot analysis: Liver and perigonadal adipose tissues (pgAT) from C57Bl/6J wild-type mice were homogenized in the presence of lysis buffer (PBS, 1% TritonX100, protease inhibitors). Protein from extracts (30 μg each sample) were resolved on 10%-acrylamide SDS-PAGE and analyzed by Western blot to measure the tissue difference in ceruloplasmin expression, normalize for the total protein content, using Goat anti-Cp antibody (Abcam, ab19171) followed by HRP conjugated secondary antibody incubation and enhanced chemiluminescence development.

Densitometric analysis: Densitometric analysis were performed using ImageJ software, and data are presented as signal ratio between pgAT/Liver for each mouse, the result shows the ceruloplasmin expression in pgAT is about two-fold increased than liver tissue homogenates.

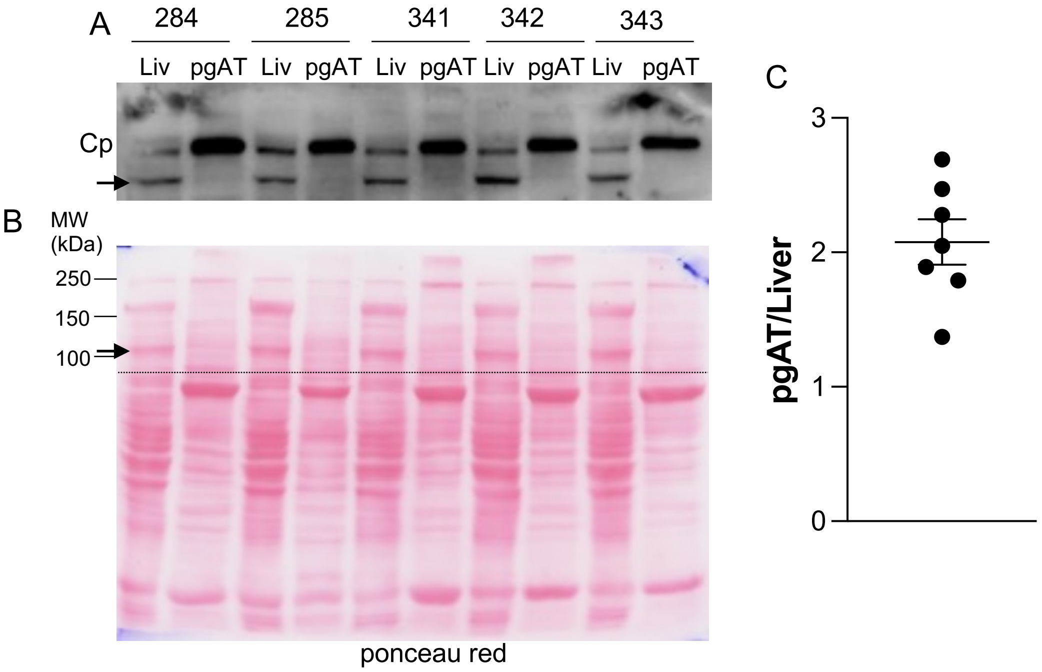

Figure S1. A) Representative images of Western blot (WB) analysis in five out of seven samples of liver and pgAT homogenates from different mouse (n=7), showed the higher expression level of Cp protein in pgAT compared to liver. **B**) Nitrocellulose filter of the representative WB showed in A stained with Ponceau-red that was used as an internal loading control for quantitative analysis. Arrows indicate the non-specific background signal in liver extracts corresponding to the large protein band visible in the ponceau red stained nitrocellulose. **C**) Quantitation of the relative Cp abundance reported as signal ratio between pgAT/Liver for each mouse.

Supplementary Material Figure S2

<u>Histological analysis of liver fibrosis by Sirius red staining</u>

Tissues from CpKO and WT mice of 10 months of age were fixed in 4% paraformaldehyde and paraffin embedded. Sirius Red staining was performed on 3 μm thick section at the Animal Histopathology facility, HSR. Samples were analyzed with Zeiss AxioImager microscope.

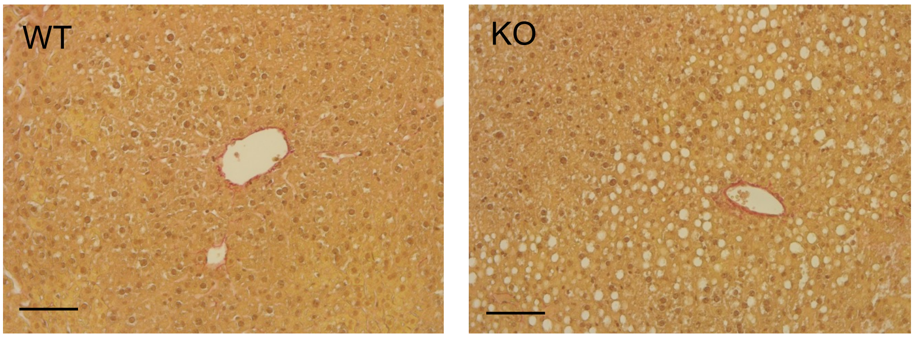

Figure S2. Representative images of Sirius Red histological staining on liver sections from 10 months-old WT and CpKO mice. Scale bars= 100 μm.

Supplementary Material Figure S3

<u>Analysis of total copper and zinc ions content in liver.</u>

Quantitative analysis of total copper and zinc metal ions content in the liver of 10 months old mice was performed by inductively coupled plasma mass spectrometry (ICP-MS) as described in Materials and Methods.

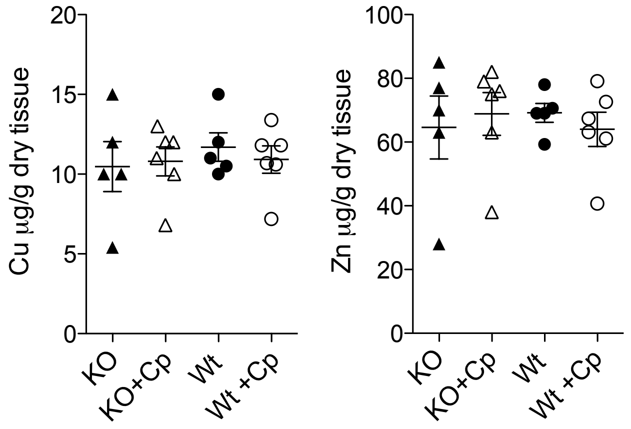

Figure S3. Copper (Cu) and zinc (Zn) metal ions evaluation in liver of ceruloplasmin-deficient (CpKO) and wild-type (WT) mice untreated or treated for 2 months with purified human ceruloplasmin (Cp) as reported in Materials and Methods. Data are reported as mean±SEM of the concentration in µg/g of dry (lyophilized) tissue of the animal groups; each dot corresponds to one animal (CpKO, WT n= 5; CpKO+Cp, WT+Cp, n=6).

Supplementary Material Figure S4

Adipose tissue accumulation in CpKO mice.

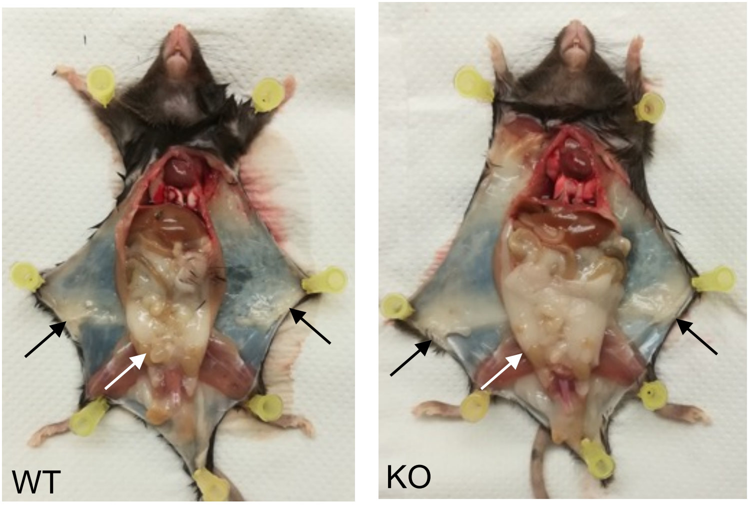

Figure S4. Representative images of perigonadal (white arrows) and sub-cutaneous (black arrows) adipose tissue accumulation in 10 months-old CpKO and WT mice.

Supplementary Material Figure S5

Analysis of adipocytes hypertrophy in CpKO mice.

To assess whether the adipose tissue mass increase at 10 months of age in CpKO mice was also paralleled by adipocytes hypertrophy, we measured the size of the adipocytes of perigonadal adipose tissue. Tissues were fixed in 4% paraformaldehyde (1 h at 4°C), transferred in 70% ethanol solution and 24 h later embedded in paraffin. Hematoxylin-eosin staining was performed on 3 μm thick section at the Animal Histopathology facility at OSR. Samples were analyzed with Zeiss AxioImager microscope. Adipocytes' size was quantified on histological images with an automated analysis protocol for area and diameter detection, set up on ImageJ software. Images were segmented by a threshold filtration to define the adipocytes boundaries, and then area and Feret's diameter for each cell was analyzed. Five images per mouse were acquired with on average 86 adipocytes/image (range 59-127), for a total of 430 adipocytes/mouse measured on average.

Even if two-ways ANOVA was not significant, the CpKO mice showed small enlargement of adipocytes size and diameter compared to WT mice suggesting slight hypertrophy of adipocytes in Cp-deficient mice.

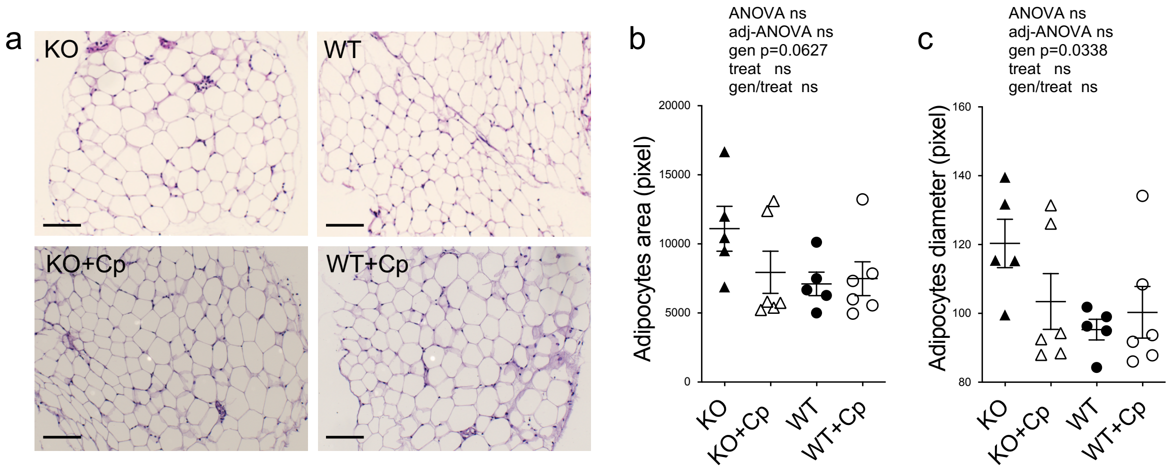

Figure S5. Adipocytes hypertrophy analysis in perigonadal adipose tissue (pgAT) of mice at 10 months of age after 2 months of treatment with intraperitoneal injection of Cp 5 µg/g, administered every 5 days (KO+Cp; WT+Cp) or saline (KO, WT). (a) Representative histological paraffin sections (3 μm thick) stained by hematoxylin-eosin of pgAT from mice. (b, c) Analysis of adipocyte size reported as pixel area (b) and Feret's diameter (c). The analysis was performed with ImageJ software. Data: mean±SEM of animal groups, each dot corresponds to one animal (CpKO, WT n= 5; CpKO+Cp, WT+Cp, n=6); statistical p values were evaluated by two-ways ANOVA and reported as value for ANOVA, value for Benjamini-Hochberg adjustment for multiple ANOVA tests related to adipose tissue features (adj-ANOVA), genotype variable (gen), treatment variable (treat) and the interaction of genotype/treatment (gen/treat). Scale bars= 100 μm.

Supplementary Material Table S1

Measured parameters

			Body weight		Liver												pgAT														sub-cAT	Serum
mouse			8m [a]	10m	Cp [a]	Oil red	Tg	F4/80	IL1ß	Fe	Cu	Zn	Hepc	Ft	Fpn	TfR1	Cp [a]	Weight	Ad size	Ad diameter	F4/80	Fe	Cu	Zn	Hepc	Ft	Fpn	TfR1	Adiponectin	Leptin	Weight	Tg
#	label	sex	(g)	(g)	OD (au)	(area %)	(µg/mg)	(area %)	(fold of WT)	(µg/g)	(µg/g)	(µg/g)	(pg/µg)	OD (au)	OD (au)	OD (au)	OD (au)	(mg)	(pixel)	(pixel)	(cells %)	(µg/g)	(µg/g)	(µg/g)	(pg/µg)	OD (au)	OD (au)	OD (au)	(ng/mg)	(ng/mg)	(mg)	(mM)
**28i**	KO	2	29.65	31.91	-0.036	3.94	26.83	8.92	5.46	2580	15	85	105.2	1.55	2.21	1.8	0.075	1272	9505	115.4	38.09	33.2	0.24	1.7	58.4	0.99	2.8	0.13	454.6	18.37	1000	0.66
**32i**	KO	2	34.92	36.67	-0.009	5.58	25.95	8.55	6.19	1616	12	77	102.2	0.54	4.12	1.2	0.072	1675	12004	131.8	32.05	24.9	0.19	1.3	8.8	2.16	3.8	0.1	225.3	29.29	971	0.57
**33i**	KO	1	43.05	47.05	0.046	6.87	34.9	10.37	6.27	1222	10	70	172.6	0.18	1.4	1.2	0.057	946	10454	115.3	49.97	6.5	0.14	1.2	27.5	1.43	3.2	0.24	133.4	37.04	1157	1.02
**4**	KO	1	48.6	53.7	-0.034	3.42	26.83	7.78	5.62	495	5.4	28	136.5	0.12	2.77	1.3	0.048	1476	16674	139.6	58.16	7.7	0.13	1.3	36.1	0.97	1.7	0.14	232.3	17.55	1152	0.78
**7**	KO	2	36	38.3	0.034	3.94	49.68	11.73	6.21	1763	10	63	175.0	1.24	1.33	1.7	0.11	1460	6887	99.6	37	2.2	0.12	2.6	47.1	1.41	3.8	0.12	485.8	33.64	1535	1.14
**2i**	KO+Cp	2	24.23	27	0.051	1.82	22.65	9.03	3.04	1400	13	79	90.2	1.56	0.84	1.4	0.155	666	5223	88	36.78	5.7	0.13	1	31.2	0.28	2.7	0.25	664.5	18.81	836	0.26
**30i**	KO+Cp	2	31.77	33.7	0.071	2.45	12.87	8.8	1.83	2239	12	82	110.6	1.33	0.2	1.6	0.181	1383	5738	136.1	34.46	16.7	0.12	1.2	2.4	0.43	3	0.15	318.2	27.35	1029	0.43
**29i**	KO+Cp	1	35.79	37.82	0.096	1.45	36.42	6.53	0.98	992	12	76	92.2	0.26	2.32	1.4	0.137	1162	5817	92.5	33.98	6.7	0.1	1.1	4.7	0.23	4	0.3	491.7	19.16	808	0.38
**5**	KO+Cp	1	43.7	46.4	0.37	1.53	39.26	7.6	2.29	479.5	6.8	38	180.2	0.07	1.39	1.4	0.619	973	5388	94.3	31.79	6.2	0.14	1.3	40.0	0.92	3	0.25	275.8	8.76	940	0.5
**2**	KO+Cp	2	35.1	37	0.522	1.96	17.45	6.97	1.11	932	11	75	128.8	0.45	1.81	1.6	0.378	722	12389	88.5	20.27	2.9	0.14	0.68	19.8	1.08	1.9	0.11	624.3	7.87	498	0.9
**6**	KO+Cp	2	31.8	35.2	0.046	1.39	26.54	6.28	0.82	1204	10	63	143.8	1.1	1.34	1.4	0.473	1054	13102	131.4	27.67	4.8	0.12	0.79	16.5	0.16	2	0.14	666.0	17.19	633	0.59
**10**	WT	1	30.5	30.9	0.337	1	4.72	6.92	1.62	160	15	69	117.7	0.12	0.38	1.2	0.412	1235	10117	99.1	18.98	6.5	0.18	1	4.5	1.45	2.5	0.07	598.2	8.50	737	0.52
**13**	WT	2	28.9	27.17	0.117	0.9	10.51	5.8	0.58	550	11	69	122.9	0.13	2.04	1.7	0.374	1165	7486	101.8	22.88	11.4	0.2	1.1	30.2	2.14	3.8	0.14	787.0	11.93	714	0.24
**14**	WT	2	30.8	29.99	0.334	0.48	10.81	7.62	1.91	951	10	78	127.6	0.18	1.28	1.6	0.263	897	5012	84.3	20.32	24.8	0.35	2.6	45.0	1.8	2.8	0.08	245.3	1.41	790	0.31
**22**	WT	1	29.8	32.6	0.467	1.66	13.63	4.73	0.34	132.4	10.5	59.3	133.8	0.06	0.39	1.7	0.476	966	6672	96.3	20.26	10.6	0.16	1.5	32.7	1.04	3.4	0.13	652.1	15.73	461	0.64
**24**	WT	2	27.4	26.2	0.278	0.76	2.27	5.65	0.59	792	12	70.6	181.8	0.13	0.98	2.1	0.453	870	6281	95	23.35	14.2	0.23	1.5	30.7	1.52	2	0.16	814.7	17.43	597	0.33
**9**	WT+Cp	1	29.3	30.53	0.33	0.59	12.87	4.5	0.24	169	13.4	63.1	111.8	0.09	1.23	1.7	0.469	663	5565	87.8	29.69	9.3	0.15	2.1	15.0	0.93	3.1	0.18	308.5	6.48	567	0.38
**11**	WT+Cp	2	28.5	28.58	0.559	0.83	12	6.18	0.4	1077	11.8	79.1	100.8	0.11	1.03	2.3	0.48	815	5987	91.8	26.38	12.6	0.43	2.2	6.1	0.91	2.5	0.11	675.0	13.52	1313	0.31
**12**	WT+Cp	2	28.7	29.55	0.335	1.22	12.54	6.43	0.85	798	10.7	72.6	118.2	0.1	0.97	1.5	0.44	1284	7326	93.7	26.34	24.1	0.23	1.5	29.1	0.73	3.7	0.21	1221.0	7.44	839	0.24
**21**	WT+Cp	1	30.2	33.5	0.186	1.98	15.09	7.5	0.75	176	10.6	61.1	112.5	0.07	1.08	2.5	0.411	920	7865	108.4	31.61	8.5	0.15	0.9	22.9	0.53	3.4	0.31	672.8	28.69	955	0.62
**23**	WT+Cp	2	28.3	26.9	0.273	1.28	16.79	5.33	0.39	517.4	7.2	40.7	139.5	0.15	0.84	2.1	0.547	1149	4953	86	39.26	9.3	0.19	1.1	60.4	0.76	4.7	0.29	971.7	15.38	589	0.57
**25**	WT+Cp	2	26.7	28	0.262	0.78	10.11	6.17	0.54	617	11.8	67.4	146.8	0.08	2	2.3	0.574	859	13217	134.2	27.53	20.9	0.73	0.8	34.6	0.46	3	0.27	615.4	7.39	690	0.55

Sex: 1= male, 2= female; 8m= 8 months; 10m= 10 months; Tg= triglycerides; Ft= ferritin; F4/80= macrophages marker; IL1ß= interleukin 1 beta; Hepc= hepcidin; Fpn= ferroportin 1; TfR1= transferrin receptor 1; pgAT= perigonadal adipose tissue; sub-cAT= subcutaneous adipose tissue;

Ad= adipocytes; OD (au)= optical density, arbitrary units;

[a] = parameter not used for PCA analysis
